# Real time magnetic resonance assessment of septal curvature accurately tracks acute hemodynamic changes in pediatric pulmonary hypertension

**DOI:** 10.1186/1532-429X-16-S1-O74

**Published:** 2014-01-16

**Authors:** Bejal Pandya, Michael A Quail, Jennifer Steeden, Andrew Taylor, Ingram Schulze-Neick, Graham Derrick, Shahin Moledina, Vivek Muthurangu

**Affiliations:** 1 Centre for Cardiovascular Imaging, UCL Institute of Cardiovascular Science, London, UK; 2 Cardiorespiratory Division, Great Ormond Street Hospital for Children, London, UK; 3 Adult Congenital Heart Disease Department, the Heart Hospital, London, UK

## Background

Non-invasive assessment of PA pressure and pulmonary vascular resistance (PVR) is vital in children with PAH. Unfortunately TR jet velocity is difficult to measure in using CMR. Therefore, we hypothesized that septal curvature (SC) assessed using CMR could be used to estimate PA pressure and track acute changes in pulmonary hemodynamics. In this study SC was compared to simultaneously acquired PA pressure and PVR in children undergoing combined cardiac catheterization and CMR

## Methods

The study included 50 children (median age of 6.6 years range 0.5-16.5 years) with either idiopathic PAH (n = 29) or PAH associated with repaired congenital heart disease (n = 17) or lung disease (n = 4). The combined CMR/catheterization was perfomed under general anesthetic and PVR was calculated using phase contrast flow and simultaneously acquired invasive PAP and wedge pressure at baseline and during vasodilation (100% 02 and 20ppm NO). SC was measured in short axis cine images at papillary muscle level using real time k-t SENSE and an in-house plugin for the OsiriX platform. A control population of 15 healthy pediatric volunteers (range 10.1-14.1 years) also underwent CMR (awake) and assessment of septal curvature (SC)

## Results

**Figure 1:**
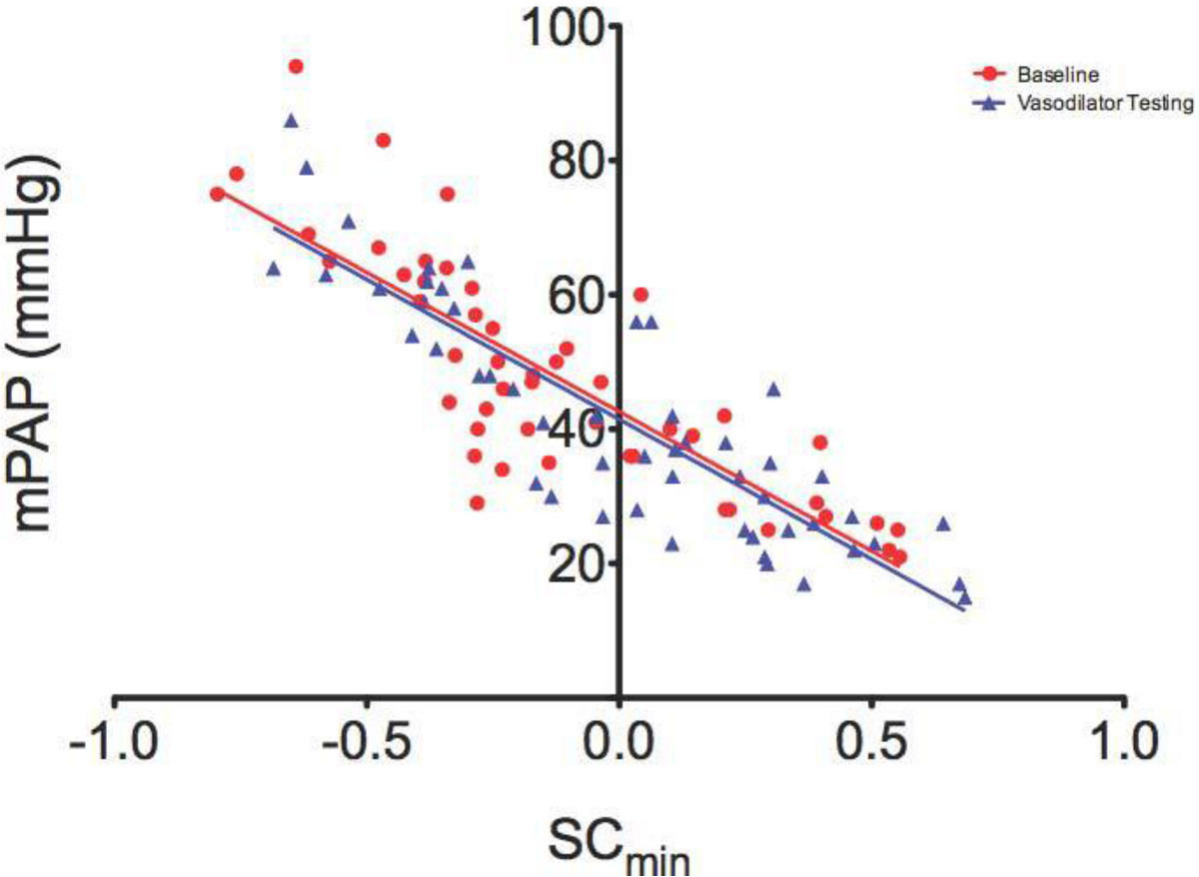
**Minimum septal curvature and mean pulmonary artery pressure (SC indicates minimum septal curvature; mPAP, mean pulmonary artery pressure; red circles are at baseline; blue triangles are during vasodilator testing)**.

**Figure 2:**
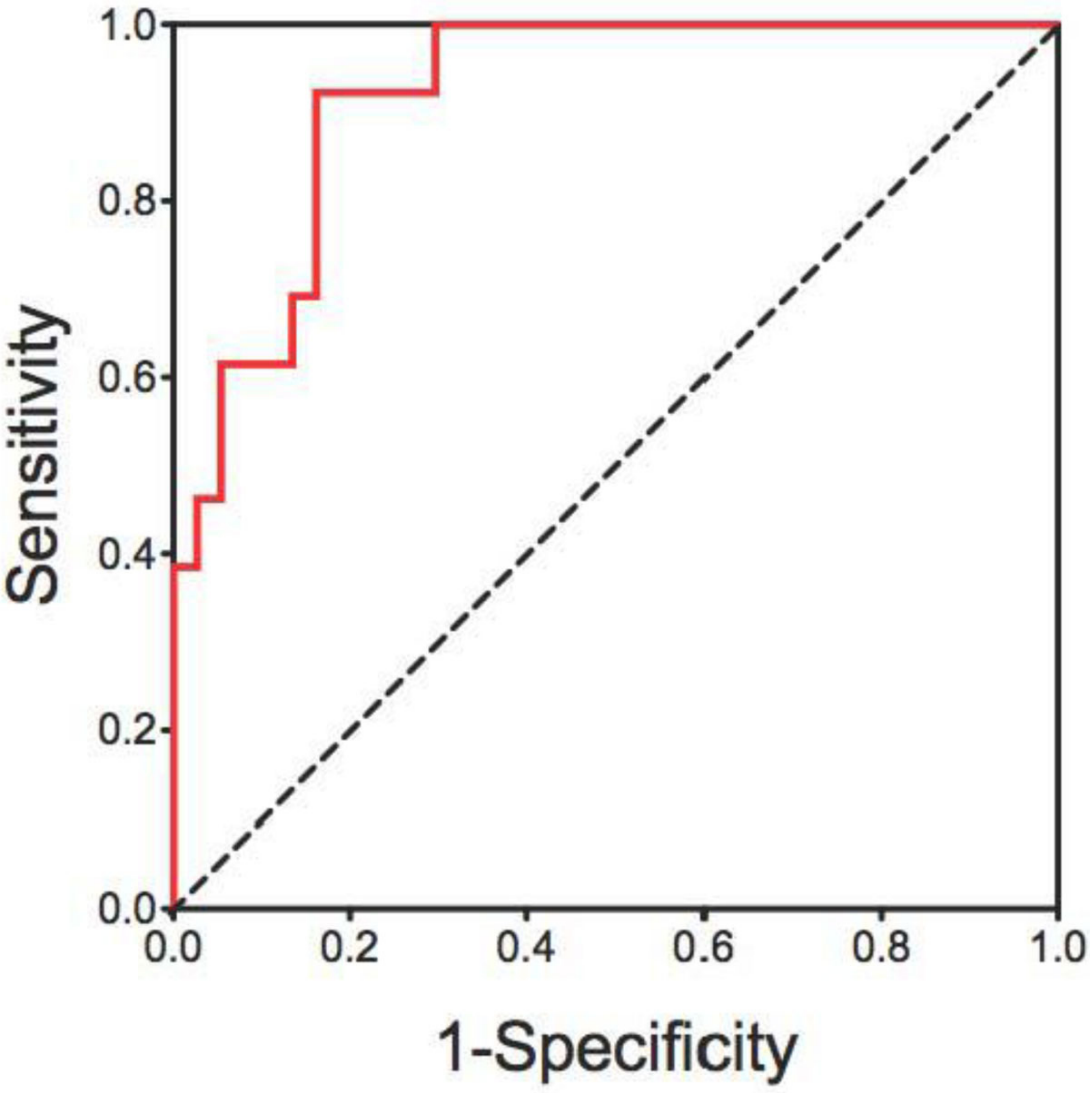
**On ROC analysis the optimal cut-off value of ΔSC for identification of vasoresponders (defined by the revised criteria) was 0.47 (AUC = 0.92 [95%CI 0.80-1.0])**. For this cut-off, the sensitivity was 68% (CI 0.51-0.99), specificity was 98% (CI 0.57-0.85), PPV was 42% (0.21-0.66) and NPV was 97% (0.81-1.0)

There was a significant difference (p < 0.05) between mean SC in the patient group (-0.12 ± 0.05, range -0.796-0.685) compared to the control group (1.02 ± 0.05, range 0.902-1.175). Importantly, there was no overlap between SC in normal controls and SC in patients. There was a strong correlation between mPAP and SC at baseline and during vasodilator testing (r = -0.84 and -0.85 respectively, p < 0.001) [Figure 1]. There was also a strong linear relationship between PVR and SC indexed to cardiac output both at baseline and during vasodilator testing (r = -0.88, r = -0.85). SC metrics moderately correlated with absolute change in mPAP and PVR (r = 0.56, r = 0.73, respectively P < 0.001). Eight patients (16%) fulfilled the revised criteria for vasoresponsivity and on ROC analysis the optimal cut-off value of ΔSC for identification of vasoresponders (defined by the revised criteria) was 0.47 (AUC = 0.92 [95%CI 0.80-1.0]). For this cut-off, the sensitivity was 68% (CI 0.51-0.99), specificity was 98% (CI 0.57-0.85), PPV was 42% (0.21-0.66) and NPV was 97% (0.81-1.0). [Figure 2]

## Conclusions

The main findings were: i) There was a significant difference in SC parameters between normal controls and children with PH; ii) SC derived metrics strongly correlated with mPAP and PVR in patients; and iii) SC derived metrics were able to track acute changes in pulmonary hemodynamics during vasodilator testing. We believe that these results show that SC metrics could be used as a non-invasive adjunct to catheter assessment in pediatric PH. Importantly, the fact the SC metrics track changes in pulmonary hemodynamics mean that they could be use to track disease progression or response to therapy. Furthermore, they could also be used to identify patients with vasoresponder status.

## Funding

British Heart Foundation and UK National Institute of Health Research.

